# Influence of surgically assisted rapid maxillary expansion on the interdental papilla height of maxillary central incisors

**DOI:** 10.1007/s00056-020-00274-y

**Published:** 2021-01-28

**Authors:** Stéphanie Noverraz, Yannick Noverraz, Tong Xi, Jan Schols

**Affiliations:** 1 Department of Orthodontics and Craniofacial Biology, Radboud University Medical Center, Philips van Leydenlaan 25, 6525 EX Nijmegen, The Netherlands; 2 Department of Dentistry, Radboud University Medical Center, Nijmegen, The Netherlands; 3 Department of Oral and Maxillofacial Surgery, Radboud University Medical Center, Nijmegen, The Netherlands

**Keywords:** Papillary recession, Jemt classification, SARME, Age, Crown morphology, Bone crest, Papilläre Rezession, Jemt-Klassifizierung, SARME, Alter, Morphologie der Krone, Aveolarkamm

## Abstract

**Purpose:**

To evaluate the influence of orthodontic treatment with surgically assisted rapid maxillary expansion (SARME) on the interdental papilla height of maxillary central incisors.

**Methods:**

In this retrospective study, patients who completed orthodontic treatment including SARME at the Radboud University Medical Center Orthodontic Department before December 2019 were included. Frontal intraoral photographs taken before (T1) and after completion of treatment (T2) were examined to determine the papilla height between the maxillary central incisors using the Jemt classification. The difference between the Jemt classification at T1 and T2 (∆Jemt) was defined as the primary outcome variable. Secondary outcome variables were gender, age, treatment duration, type of expansion appliance, maximal central diastema during expansion, pretreatment overlapping between the central incisors, gingival biotype, crown morphology and the distance between the bone crest and incisal contact point. Kappa statistics and paired t‑tests were used to determine reliability of the measurements. Pearson’s Χ^2^ test and independent t‑tests were used to compare the variables between the groups of patients with and without papillary recession. Finally, multiple logistic regression analysis was performed.

**Results:**

In all, 93 patients fulfilled the inclusion criteria and were included in the study. The Jemt score worsened for 30 patients (32%) between T1 and T2, indicating the occurrence of papillary recession. Incisal overlapping, crown morphology, small width to length ratio, increasing age and an increasing distance between crestal bone and the incisal contact point were factors associated with papillary recession.

**Conclusion:**

After orthodontic treatment including SARME, one third of patients exhibited recession of papilla height of the maxillary central incisors.

## Abstract (de)

**Ziel:**

Untersucht wurde der Einfluss einer kieferorthopädischen Behandlung mit chirurgisch unterstützten schnellen Gaumennahtweiterung (SARME) auf die Höhe der Interdentalpapille der oberen zentralen Schneidezähne.

**Methoden:**

In diese retrospektive Studie wurden Patienten eingeschlossen, die vor Dezember 2019 eine kieferorthopädische Behandlung einschließlich SARME in der kieferorthopädischen Abteilung des Radboud University Medical Center abgeschlossen hatten. Anhand von frontalen intraoralen Fotografien vor (T1) und nach Abschluss der Behandlung (T2) wurde die Papillenhöhe zwischen den oberen zentralen Schneidezähnen anhand der Jemt-Klassifikation bestimmt. Die Jemt-Klassifikation-Differenz zwischen den Werten bei T1 und T2 (∆Jemt) wurde als primäre Zielvariable definiert. Sekundäre Ergebnisvariablen waren Geschlecht, Alter, Behandlungsdauer, Art der Expansionsapparatur, maximales zentrales Diastema während der Expansion, Überlappung zwischen den zentralen Schneidezähnen vor der Behandlung, gingivaler Biotyp, Kronenmorphologie und der Abstand zwischen Knochenkamm und inzisalem Kontaktpunkt. Zur Ermittlung der Zuverlässigkeit der Messungen wurden κ‑Statistiken und gepaarte t‑Tests verwendet, der Pearson Χ^2^-Test und unabhängige t‑Tests wurden verwendet, um die Variablen zwischen den Gruppen von Patienten mit und ohne papilläre Rezession zu vergleichen. Schließlich wurde eine multiple logistische Regressionsanalyse durchgeführt.

**Ergebnisse:**

Insgesamt 93 Patienten erfüllten die Einschlusskriterien und wurden in die Studie aufgenommen. Der Jemt-Score verschlechterte sich bei 30 Patienten (32 %) zwischen T1 und T2, was auf das Auftreten einer papillären Rezession hinweist. Inzisale Überlappung, eine Kronenmorphologie, geringes Breiten-Längen-Verhältnis, ein höheres Alter und ein zunehmender Abstand zwischen krestalem Knochen und dem inzisalen Kontaktpunkt waren mit einer papillären Rezession assoziierte Faktoren.

**Schlussfolgerung:**

Nach kieferorthopädischer Behandlung einschließlich SARME wies ein Drittel der Patienten eine Rezession der Papillenhöhe der oberen zentralen Schneidezähne auf.

## Introduction

Dental esthetics is an important motivation for seeking orthodontic treatment [2]. Orthodontic treatment and combined surgical–orthodontic treatment contribute to the oral health-related quality of life (OHRQoL) as a result of the positive psychosocial impact of improved dental esthetics in combination with improved oral function [2, 12, 13, 33]. Therefore, esthetic improvement is an important outcome factor for treatment satisfaction [6, 27].

Various factors, including facial proportions, smile line, tooth position and gingival display, affect the perception of an esthetic smile [10, 31]. Laypersons assessed the presence of black triangles as the most negative factor for gingival esthetics [5]. Moreover, recession of the interdental papilla may cause phonetic problems or food impaction [28]. The interdental papilla is a fragile structure and is prone to recession. In order to preserve the interdental papilla, it is important to treat the papilla with care [28].

Surgically assisted rapid maxillary expansion (SARME) is indicated to correct transverse maxillary skeletal hypoplasia in skeletally mature patients [3, 24]. During the SARME procedure, the midpalatal suture is split. Afterwards, rapid maxillary expansion is achieved through distraction osteogenesis. The bone crest underneath the papilla between the central incisors temporarily resorbs. As the alveolar bone crest appears to be an important factor for the presence of the interdental papilla [30], the interdental papilla between the central incisors may resorb. From this point of view, SARME may be associated with papillary recession. Thus, the aim of this study was to evaluate the influence of SARME on the interdental papilla height of maxillary central incisors.

## Patients and methods

### Subjects

The study had a retrospective design. The patients’ records of the orthodontic department of the Radboud University Medical Center were reviewed. Patients were selected based on the following inclusion criteria: (1) treatment included fixed appliances in the maxilla, (2) treatment included a SARME procedure, (3) treatment was completed before December 2019, (4) presence of frontal intraoral photographs before start of the treatment (T1), (5) presence of frontal intraoral photographs after completion of treatment (T2), (6) an intact contact point between the maxillary central incisors before and after treatment. Exclusion criteria were the presence of craniofacial anomalies and severe gingival hyperplasia which made clear identification of the Jemt classification impossible.

All data were pseudonymized prior to analysis. Written informed consent was obtained from each patient. This study did not fall within the remit of the Medical Research Involving Human Subjects Act (WMO) and was carried out in accordance with the applicable legislation and policy rules, approved by the institutional review board of the Radboud University Medical Center (no. 2020-6380).

### Treatment

The treatment process started with the performance of SARME. The surgical procedure was performed by or under direct supervision of an experienced orthognathic surgeon, using the same surgical technique. Under general anesthesia an osteotomy was performed at the level of Le Fort I, with additional midline osteotomy and pterygomaxillary disjunction. The choice of the type of expansion appliance was made by agreement between the surgeon and orthodontist, taking factors such as the periodontal condition of the anchored teeth and the degree of palatal constriction into account. In patients treated with a hyrax, the tooth-borne distractor was cemented with orthodontic bands on the first premolars and first molars several days before the surgical procedure. In cases with a transpalatal distractor (TPD), the bone-borne appliance was fixated during the surgical procedure to the palatal bone with two 7 mm self-drilling screws at the level of the second premolars. Following a latency period of 1 week, the distractor was activated at an average rate of 0.5 mm/day. When the desired amount of expansion was achieved, a blocking screw was inserted in one of the boreholes of the TPD or the screw in the hyrax was covered and locked with composite. After 3 months the hyrax was replaced by a transpalatal arch on the first molars and further orthodontic treatment using fixed appliances was carried out. Purpose of the passive transpalatal arch is a prolonged retention period of the achieved maxillary arch width during the orthodontic leveling and aligning phase of treatment, up to the stage of rigid steel wires. Then the transpalatal arch is removed and diastemas are closed. Further orthodontic treatment is completed.

### Interdental papilla height classification

**Fig. 1:**
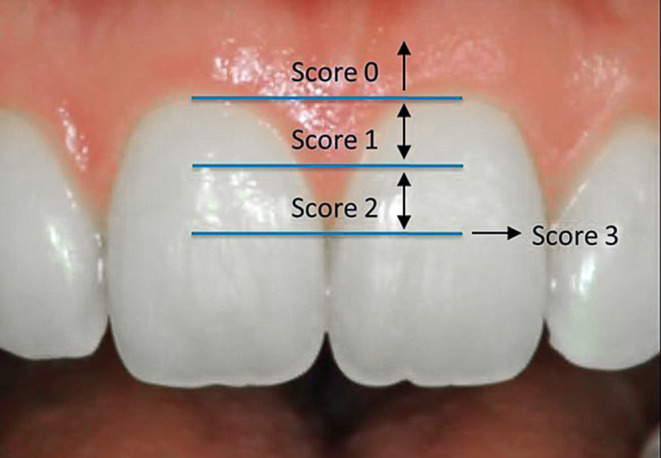
Schematic representation of the Jemt classification. Score 0: no papilla present, Score 1: less than half of the height of the papilla present, Score 2: half or more of the height of the papilla present, Score 3: the papilla fills up the entire proximal space Schematische Darstellung der Jemt-Klassifikation. Score 0: keine Papille vorhanden, Score 1: weniger als die Hälfte der Höhe der Papille vorhanden, Score 2: die Hälfte oder mehr der Höhe der Papille vorhanden, Score 3: die Papille füllt den gesamten proximalen Raum aus

Frontal intraoral photographs, taken at T1 and T2, were examined to determine the papilla height, using the index validated by Jemt [19]. The papillae were classified based on an imaginary line between the most cervical points of the maxillary central incisors and the contact point between the central incisors. The space in between was divided in two equal vertical parts (Fig. 1). The following scores were assigned to the papilla at T1 and T2:

- Score 0: No papilla is present
- Score 1: Less than half of the height of the papilla is present
- Score 2: Half or more of the height of the papilla is present
- Score 3: The papilla fills up the entire proximal space

The difference between the Jemt classification at T1 and T2 (∆Jemt) was calculated to indicate whether there was recession of the interdental papilla, a stable situation or an increase in papilla height. ∆Jemt was defined as the primary outcome variable. Based on this, the patients were divided into two groups for further subanalyses: a group of patients with papillary recession and a group without papillary recession.

### Secondary outcome variables and predictor variables

**Fig. 2:**
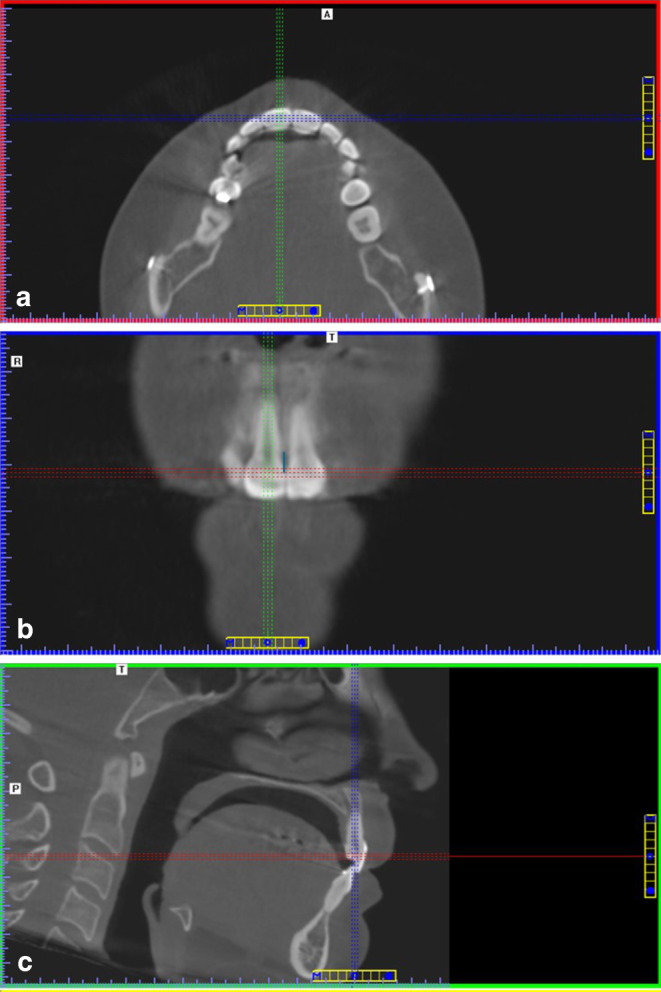
Measurement of the distance from the contact point between the maxillary central incisors to the bone crest. Tooth 11 was centered in the axial (**a**) and coronal (**b**) plane. Afterwards, the head was pitched until the pulp of tooth 11 matched the perpendicular line to the floor in the sagittal (**c**) plane. The contact point between the central incisors was established by scrolling through the images. The measurement was made in the coronal plane (*blue line*) Messung des Abstands vom Kontaktpunkt zwischen den oberen zentralen Schneidezähnen zum Alveolarkamm. Der Zahn 11 wurde in der axialen (**a**) und koronalen (**b**) Ebene zentriert. Anschließend wurde der Kopf geneigt, bis die Pulpa des 11ers mit der Senkrechten zum Boden in der sagittalen (**c**) Ebene übereinstimmte. Der Kontaktpunkt zwischen den zentralen Schneidezähnen wurde durch Scrollen durch die Bilder ermittelt. Die Messung erfolgte in der koronalen Ebene (*blaue Linie*)

- Gender: patient’s files were used.
- Age at time of SARME: patient’s files were used.
- Treatment duration: patient’s files were used.
- Type of used expansion appliance (tooth-borne hyrax or bone-borne TPD): patient’s files were used.
- Maximal central diastema during expansion: patient’s files were used.
- Pretreatment overlapping between the mesial sides of maxillary incisors: a categorical classification was made regarding the presence or absence of overlapping. Intraoral photographs at T1 were used for the assessment.
- Gingival biotype: a categorical classification was made into thin, normal or thick biotype. Intraoral photographs at T1 were used for the assessment.
- Crown morphology: assessment was made using (1) the ratio between crown width and crown length was determined (W:L). Crown width was considered as the distance between the approximal surfaces of adjacent teeth. The crown length was considered as the distance between the incisal edge and the gingival margin or the cement–enamel junction if visible [20, 25]. (2) A categorical crown morphology (CM) classification was made into oval, triangular or square teeth.
- The distance between the bone crest and the incisal contact point (CP‑B T1, CP‑B T2) and change in distance over time (∆CP-B): cone beam computed tomography (CBCT) images were used (Fig. 2). In most cases, CBCT images were made before the start of the surgical–orthodontic treatment and 1 or 2 years after final surgery. The CBCT interval was matched as much as possible with the treatment duration, in order to have images closely related to the situation at T1 and T2.

### Statistical analysis

The data were analyzed by using IBM SPSS Statistics for Windows, version 25.0 (IBM Corp., Armonk, NY, USA). All measurements were performed by one observer and repeated after 2 weeks to test intraobserver reliability. A second observer repeated the measurements to test interobserver reliability. The reliability was determined using kappa statistics for categorical variables and paired t‑test for continuous variables.

The incidence of each Jemt score at T1 and T2 was calculated. The difference between T1 and T2 was calculated and pooled into an occurrence and nonoccurrence group of papilla recession. Pearson’s Χ^2^ test and independent t‑test were used to compare the categorical and continuous variables between the groups of patients with and without papillary recession.

**Fig. 3:**
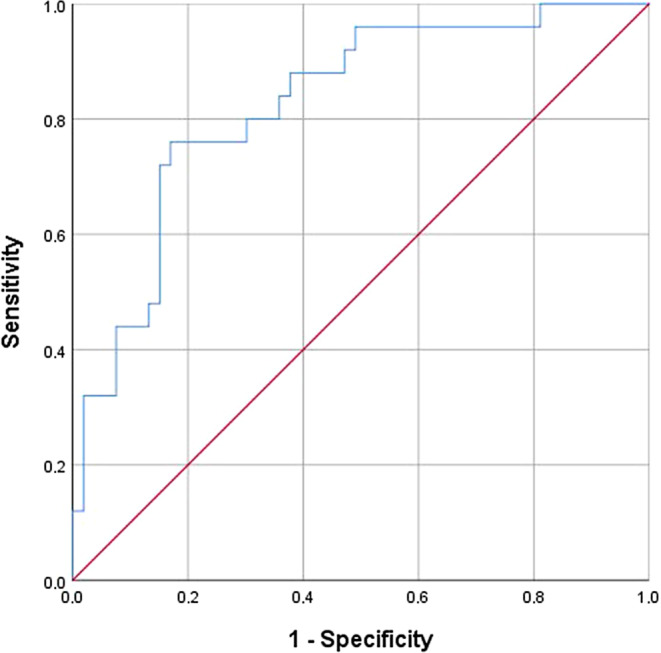
Receiver operating characteristic (ROC) curve of the multiple regression model in Table 6. AUC (area under the curve) = 0.83 ROC(“receiver operating characteristic”)-Kurve des multiplen Regressionsmodells in Tab. 6. AUC(Fläche unter der Kurve>) = 0,83

Multiple logistic regression analysis was used to identify associations between various significant variables with the occurrence of papilla recession as the dependent variable and age, W:L and ∆CP‑B as predictor variables. A receiver operating characteristic (ROC) curve was calculated to quantify the probability (fitting) of the model (Fig. 3).

## Results

In all, 93 patients (33 males and 60 females) were included in this study. At the time of SARME, the mean age was 29.00 ± 11.17 years. The mean duration of orthodontic treatment was 2.79 ± 0.76 years. The mean interval between the preoperative and postoperative cone beam computed tomography (CBCT) was 2.66 ± 0.90 years. The included patients all had closed central diastemas at T2. All demographic and clinical data of patients are presented in Table 1 (categorical variables) and Table 2 (continuous variables).

**Table 1:** Descriptive statistics of the categorical variables Deskriptive Statistik der kategorialen Variablen

Categorical variable (abbreviation)	Number	%
*Gender (Gender)*		
Male	33	35.5
Female	60	64.5
*Expansion appliance (Appliance)*		
Transpalatal distractor	54	58.1
Hyrax	39	41.9
*Overlapping between the maxillary central incisors at T1 (Overlapping)*		
No overlapping	70	75.3
Overlapping	23	24.7
*Biotype (Biotype)*		
Thin	12	12.9
Normal	70	75.3
Thick	11	11.8
*Crown morphology (CM)*		
Oval	65	69.9
Triangular	11	11.8
Square	17	18.3

**Table 2:** Descriptive statistics of the continuous variables Deskriptive Statistik der kontinuierlichen Variablen

Variable (abbreviation)	Number of subjects	Mean	SD	Min	Max
Age at time of SARME (Age)	93	29.00	11.17	13.16	64.62
Treatment duration (Duration)	90	2.79	0.76	1.42	4.99
CBCT interval (CBCT interval)	78	2.66	0.90	0.22	5.00
Width:length ratio (W:L)	93	0.83	0.09	0.66	1.11

*SD* standard deviation, *Min* minimum, *Max* maximum, *CBCT* cone beam computed tomography

### Intra- and interobserver reliability

The intraobserver reliability for the categorical variables was excellent (overlapping: kappa 0.97; biotype: kappa 0.80; Jemt T1: kappa 0.94; Jemt T2: kappa 0.93; CM: kappa 0.79), indicating a substantial consistency of the measurements. The intraobserver reliability for the continuous variables was good as well (CP‑B T1: reliability 0.81, Duplicate Measurement Error (DME) 0.49, systematic difference 0.06, *p* = 0.43; CP‑B T2: reliability 0.87, DME 0.53, systematic difference 0.03, *p* = 0.73; W:L: reliability 0.99, DME 0.01, systematic difference −0.01, *p* = 0.00).

The kappa values to assess the interobserver reliability for categorical variables indicated consistent measurements between the two examiners (overlapping: kappa 0.80; biotype: kappa 0.47; Jemt T1: kappa 0.77; Jemt T2: kappa 0.78), except for crown morphology (kappa 0.35). The interobserver reliability for CP‑B T1 and CP‑B T2 was acceptable (CP‑B T1: reliability 0.73, DME 0.57, systematic difference 0.12, *p* = 0.17; CP‑B T2: reliability 0.76, DME 0.71, systematic difference 0.32, *p* = 0.01). The interobserver reliability for W:L ratio was excellent (reliability 0.96, DME 0.02, systematic difference 0.02, *p* = 0.00).

### Periodontal changes of the maxillary central papilla

The morphology of the maxillary central papilla in terms of Jemt classification is shown in Table 3.

**Table 3:** Distribution of subjects according to the Jemt classification before treatment (T1) and after completion of treatment (T2) Verteilung der Probanden nach der Jemt-Klassifikation vor der Behandlung (T1) und nach Abschluss der Behandlung (T2)

Jemt classification	Subjects at T1, *N* (%)	Subjects at T2, *N* (%)
0	1 (1.1)	3 (3.2)
1	2 (2.2)	9 (9.7)
2	18 (19.4)	31 (33.3)
3	72 (77.4)	50 (53.8)
Total	93 (100)	93 (100)

*0:* no papilla present, *1:* less than half of the height of the papilla present, *2:* half or more of the height of the papilla present, *3*: the papilla fills up the entire proximal space

In all, 72 patients were classified with Jemt score 3 at T1, indicating a complete papilla fill. At T2, 50 out of 72 patients had a complete papilla fill. An overall decrease in the mean Jemt score was observed between T1 (2.73) and T2 (2.38), indicating a general trend of papilla recession (*p* < 0.01). The changes in Jemt classification between T1 and T2 and the pooled Jemt changes (papilla with recession versus papilla without recession) over time are presented in Table 4. As displayed in the tables, the Jemt score worsened for 30 patients between T1 and T2, mostly with a decrease of one class (24 of 30 patients, 80%).

**Table 4:** Changes in Jemt classification between prior to treatment (T1) and after completion of treatment (T2) Änderungen in der Jemt-Klassifikation zwischen vor der Behandlung (T1) und nach Abschluss der Behandlung (T2)

Papilla change between T1 and T2 according to the Jemt classification	Subjects *N* (%)	∆Jemt	Frequency (%)
Papilla with recession	30 (32.3%)	−2	6 (6.5)
		−1	24 (25.8)
Papilla without recession	63 (67.7%)	0	60 (64.5)
		+1	3 (3.2)

A comparison of various categorical patient-specific factors between patients who displayed papilla recession and who did not exhibit papilla recession is presented in Table 5. The Χ^2^ tests revealed that the number of patients with overlapping central incisors (*p* = 0.02) in the group with papilla recession was significantly higher than in the group without papilla recession. The crown morphology was significant different between the groups as well (*p* = 0.04).

**Table 5:** Comparison of papilla with recession and papilla without recession for categorical variables Vergleich zwischen Papille mit Rezession und Papille ohne Rezession für kategoriale Variablen

Variable		Papilla with recession *N*	Papilla without recession *N*	Pearson’s Χ^2^	*P*-value
Gender	Male (%)	9 (27.3)	24 (72.7)	0.58	0.45
	Female (%)	21 (35.0)	39 (65.0)		
Appliance	TPD (%)	15 (27.8)	39 (72.2)	1.18	0.28
	Hyrax (%)	15 (38.5)	24 (61.5)		
Overlapping	No overlapping (%)	18 (25.7)	52 (74.3)	5.55	0.02*
	Overlapping (%)	12 (52.2)	11 (47.8)		
Biotype	Thin (%)	4 (33.3)	8 (66.7)	0.14	0.93
	Normal (%)	23 (32.9)	47 (67.1)		
	Thick (%)	3 (27.3)	8 (72.7)		
CM	Oval (%)	25 (38.5)	40 (61.5)	6.64	0.04*
	Triangular (%)	4 (36.4)	7 (63.6)		
	Square (%)	1 (5.9)	16 (94.1)		

*CM* crown morphology, *N* number of patients, *TPD* transpalatal distractor

*Significant, Pearson’s Χ^2^ was used

Independent t*-*test was performed to compare the age, treatment duration, distance between crestal bone and incisal contact point, and width/length ratio of central incisor between the group with and without papilla recession (Table 6). Patients with papilla recession were significantly older, the distance between the incisal contact point and crest of bone was significantly more increased and the W:L ratio was significantly smaller (indicating a more narrow crown morphology) compared to the patients without papilla recession.

**Table 6:** Comparison of papilla with recession and papilla without recession for continuous variables Vergleich zwischen Papille mit Rezession und Papille ohne Rezession für kontinuierliche Variablen

Variable	Papilla with recession Mean (SD)	Papilla without recession Mean (SD)	Mean difference (SE)	*P*-value	95% CI of the difference [lower;upper]
Age	34.30 (10.64)	26.48 (10.59)	7.82 (2.35)	0.00*	[3.15; 12.50]
Duration	2.89 (0.84)	2.70 (0.80)	0.19 (0.18)	0.29	[−0.17; −0.55]
CP‑B T1	4.27 (1.17)	4.74 (1.09)	−0.47 (0.26)	0.08	[−1.00; 0.54]
CP‑B T2	6.30 (1.50)	5.68 (1.41)	0.62 (0.35)	0.08	[−0.07; 1.31]
∆CP‑B	2.02 (1.41)	1.00 (1.64)	1.03 (0.38)	0.01*	[0.26; 1.79]
W:L	0.80 (0.07)	0.85 (0.10)	−0.05 (0.02)	0.02*	[−0.88; −0.01]

*CP‑B T1* distance between the bone crest and the contact point at T1, *CP‑B T2* distance between the bone crest and the contact point at T2, *∆CP‑B* change in distance over time, *W:L* ratio between crown width and crown length, *SD* standard deviation, *SE* standard error, *CI* confidence interval

*Significant

Since the maximal central diastema immediately after SARME was recorded in only 20 out of 93 patients’ files, it was inappropriate to statistically test the influence of this factor in a reliable way. Thus, this variable was excluded from statistical analysis.

A multiple logistic regression model was constructed to predict the probability of papilla recession (Table 7). All variables were significant predictors (*p* < 0.05). After controlling for the other variables in the model, the odds for papilla recession increased 10% for every year increase in age, increased 50% for every millimeter increase in ∆CP‑B between T1 and T2 and decreased 10% ([1 / 0.91] × 100%) for every 0.01 increase in width/length ratio. The area under the curve (AUC) of the model was 0.83, indicating an excellent fitting (predicting probability) of this model (Fig. 3).

**Table 7:** Multiple logistic regression analysis for the occurrence of papilla recession (dependent variable) and age, W:L and ∆CP‑B as predictor variables Multiple logistische Regressionsanalyse für das Auftreten von Papillenrezessionen (abhängige Variable) und Alter, W:L und ∆CP‑B als Prädiktorvariablen

Variable	OR	95% CI [lower;upper]	*P* value
Age	1.10	[1.04; 1.16]	0.00*
∆CP‑B	1.50	[1.02; 2.20]	0.04*
(W:L) × 100	0.91	[0.84; 0.98]	0.01*

*∆CP‑B* change in distance over time, *W:L* ratio between crown width and crown length, *OR* odds ratio, *CI* confidence interval

*Significant

## Discussion

The results of this study demonstrated that papillary recession between the maxillary central incisors is a common finding in orthodontic patients who underwent SARME. In all, 32.3% of the patients exhibited a decreased Jemt score after orthodontic treatment including SARME, indicating recession of the central papilla.

The importance of the interdental papillae for smile esthetics was underlined in earlier studies [16, 29]. Furthermore, the actual visibility of a papilla recession should also be taken into account in the assessment of smile esthetics. Former research demonstrated that 84–91% of the subjects revealed interdental papillae upon smiling [17, 18]. Even in 87% of the subjects with a low gingival smile line, their interdental papillae was visible [17]. In these cases, the presence of a central papilla recession was associated with a decrease in smile esthetics [5]. Therefore, a recession of the interdental papilla should be assessed as a clinically relevant outcome following orthodontic treatment.

Although papillae are mostly visible during smiling, age and gender affect the smile line and thus the recession visibility. Females often have a higher gingival smile line compared to males; and with increasing age, the upper lip length increases, reducing the amount of visible gingiva in the upper incisal region [17, 23, 32]. The mean age was significantly higher in patients who exhibited papilla recession compared to patients without papilla recession, 34.3 years versus 26.5 years, respectively. This is in line with former studies [8, 11, 18]. Although the visibility of recessions decreases with age, the risk of developing recessions increases with increasing age. This particularly holds true for patients undergoing SARME as a part of their orthodontic treatment as they are mostly adolescent or adult patients. Besides this, a systematic review [2] demonstrated that the main motives for seeking orthognathic treatment were increased self-confidence, better oral function and improvement in appearance. Because papilla recession can negatively affect appearance, clinicians should manage patient expectations before the start of orthognathic treatment and make patients aware of this prior to SARME.

As 32% of the patients exhibited central papillary recession following SARME, factors that are associated with this phenomenon were explored. In periodontology, the vertical distance from the incisal contact point to the crest of the alveolar bone is associated with the presence or absence of papillary recession. Studies have reported a 98% presence of the interdental papilla when this distance was 5 mm or less, 56% presence at a distance of 6 mm and only 27% presence at a distance of 7 mm [30]. More recent studies are in agreement with this finding [4, 11, 20, 22]. This study revealed that the height between crestal alveolar bone and the incisal contact point before and after treatment including SARME in the group with recession was lower compared to the group with recession, though this difference was just short of reaching statistical significance. The lack of significance can be explained by the measurement methods used. Since the present study was performed retrospectively, it was not possible to perform bone sounding to measure this distance in the manner as it was done in other research [30]. Nevertheless, the distance between crestal alveolar bone and the incisal contact point was of importance with regard to papillary recession as patients with recession displayed a significantly larger increase in this distance over time compared to patients without recession, 2.0 and 1.0 mm, respectively. Due to the SARME procedure, the bone crest underneath the papilla between the central incisors showed a tendency to resorb temporarily. Several studies evaluated the ossification of the midpalatal suture after SARME. Whereas bone density in the midpalatal suture increased after SARME, the density after 7 months was still only 48–81% of its initial density [14, 26]. This indicated that bone formation and ossification were not yet completed. Numerous factors that could have an effect on the formation of new bone were reviewed [15]. Since the position of the crest of bone is important for the presence of the central papilla, the factors influencing bone formation in general, may also have influenced the papilla formation after SARME.

In this study, 52.2% of the patients with mesial overlapping of the central incisors developed recession of the central papilla. In the literature, black triangle development after orthodontic alignment was found in 41.9% of patients with overlapping incisors [7]. Depending on the type of tooth movement needed to align crowded incisors, the risk of developing black triangles varies. Labial movement of central incisors was more prone to papilla recession compared to palatal movement [21]. Thus, the overlapping of central incisors alone, irrespective of SARME, can be considered as a risk factor for papillary recession.

Another factor which may affect the presence or absence of papilla recession may be the crown morphology [7, 22]. Usually the contact point between triangular shaped crowns is positioned more incisally compared to square and oval shaped crowns. This increases the distance between the incisal contact point and alveolar bone crest. As mentioned before, this increases the risk of developing papillary recession [30]. In square teeth the contact area is longer and as a consequence this requires less of the papilla to fill the embrasure, explaining the increased likelihood of complete papillary fill [9]. This study showed likewise results with only 5.9% of teeth with a square morphology displaying recession of the central papilla. Another way to evaluate the crown morphology is through the width:length (W:L) ratio. The mean W:L ratio for the papillary recession group and the group without recession were 0.80 ± 0.07 and 0.85 ± 0.10, respectively. These findings are comparable to the ratios reported in the literature (0.79 ± 0.06 vs. 0.88 ± 0.07) [20]. The significant larger W:L ratio in patients without papilla recession indicates a more square crown shape, which is in agreement with the results of the categorical allocation in crown morphology.

As described above, we were able to identify five significant variables affecting the presence of papilla recession. Using these variables, multiple logistic regression models were established to predict the probability of papilla recession. Because the sample size was limited, it was only possible to include three variables in the final model. The strongest model (AUC = 0.83) showed that age, ∆CP‑B and W:L ratio were independent and significant predictors for papilla recession after orthodontic treatment including SARME. These findings may aid clinicians to understand the risks of developing papillary recession.

Previous studies identified increased gingival thickness also to be associated with complete papilla fill [11, 20]. Although it seems obvious that a thick gingival biotype is more resistant to damage, the results of the earlier studies are inconsistent [22]. In our study, we were not able to identify a thin biotype as a risk factor. This may be due to the difficulty in defining the biotype on intraoral photographs. Although this method is commonly used, its reliability is questionable [1]. It may be possible to determine the gingival biotype in a more sensitive way during clinical examination in future prospective studies, rather than depending on photographs like in this retrospective study. In a prospective study design, it will also be possible to record the maximal central diastema after expansion in all patients. In this way, the potential influence of this variable could be investigated as well. The absence of a control group made it more difficult to determine for what part the SARME contributed to recession of the papilla. However, CBCT images were rarely available in nonsurgical orthodontic patients, causing lack of comparable information about the distance between bone crest and the incisal contact point. Moreover, a control group with nonsurgical expansion treatment seems most suitable. However, nonsurgical expansion is only used in skeletally immature, and thus young patients. If the distance between bone crest and the incisal contact point and age are considered as important factors, a control group without comparable values of these variables may bias the results. It can be concluded that it is difficult to find a suitable, nonbiased control group.

## Conclusion

It can be concluded that 32% of the patients after orthodontic treatment including SARME exhibited recession of the interdental papilla height of maxillary central incisors. Within the limitations of the present study, it was found that factors such as age, width:length ratio of the clinical crown, the change in the distance from alveolar bone crest to incisal contact point, and an overlapping between the maxillary central incisors were associated with papillary recession. The findings of this retrospective study may be useful for clinicians to inform patients better about the risk of papillary recession prior to SARME.
